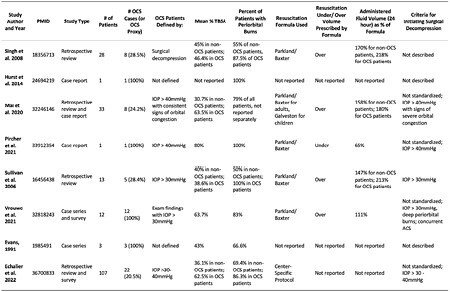# 79 Diagnosis and Management of Orbital Compartment Syndrome in Burn Patients —a Systematic Review

**DOI:** 10.1093/jbcr/irae036.078

**Published:** 2024-04-17

**Authors:** Nathan Makarewicz, David Perrault, Clifford C Sheckter

**Affiliations:** Stanford University, Stanford, California; Stanford/Santa Clara Valley Medical Center, San Jose, CA; Stanford University, Stanford, California; Stanford/Santa Clara Valley Medical Center, San Jose, CA; Stanford University, Stanford, California; Stanford/Santa Clara Valley Medical Center, San Jose, CA

## Abstract

**Introduction:**

Orbital compartment syndrome (OCS) is a recognized yet poorly understood problem in the acute management of major burns. Proposed risk factors including large volume resuscitation and deep burns to the orbital region, though OCS has been described in less severe burns. Diagnosis remains inconsistent in terms of pressure thresholds, and management is controversial regarding indications for surgical decompression. A systematic review is necessary to understand OCS in greater detail and guide management.

**Methods:**

A systematic review of the literature discussing OCS in burn patients was performed according to Preferred Reporting Items for Systematic Reviews and Meta-Analyses (PRISMA) guidelines. PubMed, Cochrane Library, and Embase were searched from inception of literature until June 2023. All primary literature publications were considered. Animal studies were excluded. Study quality was assessed using two validated scoring systems.

**Results:**

303 unique articles were identified of which 8 met inclusion criteria. All studies were retrospective. 5/8 studies measured intraocular pressure. 2 studies defined OCS at IOP>40mmHg, 2 studies defined OCS at IOP>30mmHg, and one defined OCS at IOP>30-40mmHg. A total of 60 unique cases with OCS were reported. The weighted mean total body surface area (TBSA) of burn was 58%. The weighted mean percentage of patients with orbital burns was 85%. The mean weighted 24-hour resuscitation volume was 6.64cc/kg/TBSA. Surgical treatment was always a lateral canthotomy, and some authors added cantholysis. When post-canthotomy pressures were taken, they normalized in every case. Long-term outcomes such as vision were not consistently reported. Further, reconstructive management was not described. Mortality ranged in the studies between 8-67%. The methodological index for non-randomized studies (MINORS) rated three studies as good, one as fair, and four as poor.

**Conclusions:**

The most common patient with OCS is a large surface area (>55% TBSA) burn with facial involvement undergoing resuscitation that exceeds 6cc/kg/TBSA. A precise decompressive threshold remains conflicted though all studies agree on a 30-40mmHg. Surgical decompression consisted of lateral canthotomy with the addition of cantholysis if pressures did not normalize.

**Applicability of Research to Practice:**

Burn practitioners should be aware of risk factors for OCS and measure IOP in any large TBSA burn (i.e. >40% TBSA) with facial involvement undergoing resuscitation exceeding 4cc/kg/TBSA. Lateral canthotomy should be performed in timely fashion with confirmation of normalization of IOP. Persistently elevated IOP should be treated with cantholysis.